# Cryotherapy for partial gland ablation of prostate cancer: Oncologic and safety outcomes

**DOI:** 10.1002/cam4.5692

**Published:** 2023-02-12

**Authors:** Mamdouh N. Aker, Wayne G. Brisbane, Lorna Kwan, Samantha Gonzalez, Alan M. Priester, Adam Kinnaird, Merdie K. Delfin, Ely Felker, Anthony E. Sisk, David Kuppermann, Leonard S. Marks

**Affiliations:** ^1^ Department of Urology David Geffen School of Medicine at University of California Los Angeles California USA; ^2^ Avenda Health Culver City California USA; ^3^ Department of Urology University of Alberta Edmonton Alberta USA; ^4^ Department of Radiology David Geffen School of Medicine at University of California Los Angeles; ^5^ Department of Pathology David Geffen School of Medicine at University of California Los Angeles California USA

**Keywords:** Prostate cancer, cryotherapy, focal therapy

## Abstract

**Background:**

Partial gland ablation (PGA) is a new option for treatment of prostate cancer (PCa). Cryotherapy, an early method of PGA, has had favorable evaluations, but few studies have employed a strict protocol using biopsy endpoints in men with clinically significant prostate cancer (csPCa).

**Methods:**

143 men with unilateral csPCa were enrolled in a prospective, observational trial of outpatient PGA‐cryotherapy. Treatment was a 2‐cycle freeze of the affected prostate part. Participants were evaluated with MRI‐guided biopsy (MRGB) at baseline and at 6 months and 18 months after treatment. Absence of csPCa upon MRGB was the primary endpoint; quality‐of‐life at baseline and at 6 months after treatment was assessed by EPIC‐CP questionnaires in the domains of urinary and sexual function.

**Results:**

Of the 143 participants, 136 (95%) completed MRGB at 6 months after treatment. In 103/136 (76%), the biopsy revealed no csPCa. Of the 103, 71 subsequently had an 18‐month comprehensive biopsy; of the 71 with 18‐month biopsies, 46 (65%) were found to have no csPCa. MRI lesions became undetectable in 96/130 (74%); declines in median serum PSA levels (6.9 to 2.5 ng/mL), PSA density (0.15 to 0.07), and prostate volume (42 to 34cc) were observed (all *p* < 0.01). Neither lesion disappearance on MRI nor PSA decline correlated with biopsy outcome. Urinary function was affected only slightly and sexual function moderately.

**Conclusion:**

In the near to intermediate term, partial gland ablation with cryotherapy was found to be a safe and moderately effective treatment of intermediate‐risk prostate cancer. Eradication of cancer was better determined by MRI‐guided biopsy than by MRI or PSA.

## Graphical abstract

In the near to intermediate term, partial gland ablation with focal cryotherapy was found to be a moderately effective and relatively safe treatment for men with intermediate risk prostate cancer. We also found that treatment success, that is, eradication of the cancer, is best determined by MRI‐guided biopsy, rather than by PSA levels or MRI findings.

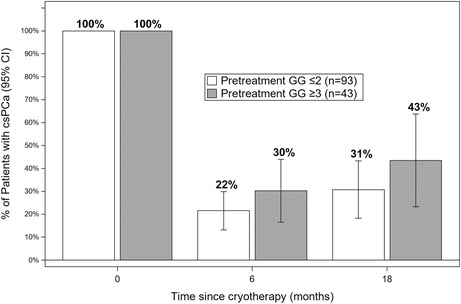

**Lay summary**

Partial gland ablation with focal cryotherapy was found to be a moderately effective and relatively safe treatment for men with intermediate risk prostate cancer. We also found that treatment success, that is, eradication of the cancer, is better determined by MRI‐guided biopsy than by PSA levels or MRI findings. The study was prospective, of intermediate term, and without a comparator.

## 1 INTRODUCTION

Cryotherapy, the focal application of ultra‐cold temperature, is the original modality for partial gland ablation of prostate cancer (PCa). Onik and colleagues described the freezing of unilateral PCa using argon gas in 2002.^1^ Subsequently, these authors introduced the term “male lumpectomy”,^2^ referring to the localized ablation of a tumor in the prostate, analogous to Fisher's lumpectomy for breast cancer.^3^ Interest in focal therapy of PCa, that is, partial gland ablation (PGA), may be dated from that time and is now increasing rapidly.^4^ However, randomized trials of PGA‐cryotherapy (or any other form of PGA) are not currently available.^5^

The mechanisms of cryoablation and the evolution of prostate cryotherapy have been reviewed in detail by Theodorescu.^6^ Numerous cohort studies over the past decade suggest that PGA with cryotherapy may be safe and effective.^7^, ^8^, ^9^, ^10^, ^11^, ^12^, ^13^, ^14^ A report from an online registry, also supporting the treatment, included data from 829 men.^15^ However, many of the studies are retrospective, and follow‐up biopsy—which provides the most compelling evidence of treatment outcome—is often reported only “for cause,” for example, when serum PSA levels increase. Moreover, in many of the previous studies, men with low‐grade tumors (Gleason Score of 6) were included. Thus, despite the encouraging reports, PGA cryotherapy of PCa is currently considered investigational.^16^, ^17^ Prospective studies of PGA cryotherapy that include biopsy per protocol before and after treatment, especially MRI‐guided biopsy (MRGB), would therefore be of interest.

To obtain further information regarding the safety and effectiveness of focal cryotherapy for PCa, an observational trial was begun in 2017. The data from that trial, presented herein, differ from other trials in which cryotherapy was used to achieve PGA: A registered protocol was employed prospectively; per‐protocol biopsies (targeted, tracked, and systematic) were obtained before and after treatment at near‐ and intermediate‐term time points; all biopsies were guided by concomitant MRI using MRI/US fusion (MR‐guided biopsy, MRGB); all patients had clinically significant PCa (csPCa); and a detailed evaluation of sexual and urinary after‐effects was included. A partial analysis of early patients in the trial was included in two previous reports.^18^, ^19^

## 2 MATERIALS AND METHODS

### 2.1 Design

**FIGURE 1:**
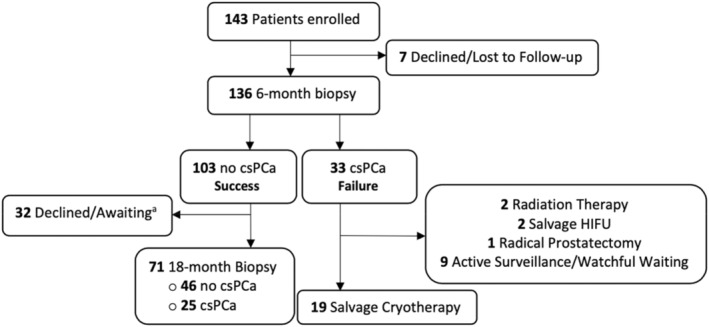
FLOW DIAGRAM. Diagram shows flow of patients from enrollment to completion of endpoints. 143 men with clinically significant prostate cancer (csPCa) were enrolled. csPCa is defined as GG1 >6 mm (*N* = 5) or any amount of PCa >GG2 (*N* = 138). Success is defined as absence of any csPCa on MRI‐guided biopsy. Small amounts of GG1 (<6 mm) were regarded as incidental findings and not considered herein. Among the 25 patients with csPCa at 18 months, 10 are continuing active surveillance (GG2), 5 underwent radical prostatectomy, 3 had radiation therapy, and 7 are undergoing repeat ablation therapy.

The study is an observational trial involving a uniform cohort of men with csPCa, treated by PGA‐cryotherapy between February 2017 and October 2021 at UCLA Medical Center. All data were acquired prospectively in a registered protocol (NCT03503643). Data were incorporated into a single‐center registry, the UCLA fusion database, which was established in 2009.^20^ Upon enrollment, all patients provided an informed consent authorized in advance by a UCLA IRB (ID No. 17‐001084). The study was conducted and herein described in accordance with the STROBE guidelines (Strengthening the Reporting of Observational Studies in Epidemiology).^21^ A flow diagram is shown in Figure 1.

### 2.2 Subjects

143 men with unilateral csPCa, who were diagnosed by MRGB at UCLA Medical Center during the 4‐year interval, were subjects of the study. Inclusion criteria included unilateral csPCa, age 40–85 years; PSA <20 ng/mL; prostate volume <70cc; and a life expectancy of at least 10 years. csPCa was defined as PCa ≥GG2 (*n* = 138) or high‐volume GG1 (cancer core length ≥6 mm, *n* = 5).^22^ Patients with medical contraindications to MRI, biopsy, or anesthesia, or who had previous treatment for prostate cancer were excluded. None received androgen deprivation therapy. PGA was elected by patients after a counseling session, during which surgery, radiation, high‐intensity focused ultrasound (HIFU), and active surveillance were also discussed. Of the 143 men enrolled, 128 had GG2 and GG3 lesions (Table 1), which represented 15% (128/843) of all men diagnosed with intermediate risk PCa during the 4‐year study interval.

**TABLE 1:** Baseline characteristics (*n* = 143).

Age, years, mean (SD)	68.3 (7.3)
Race	
African American	12 (8%)
Asian	4 (5%)
Caucasian	113 (79%)
Other^a^	13 (8%)
Prostate volume, cc	
≤40	69 (48%)
41–55	41 (29%)
>55	33 (23%)
ROI characteristics	
PI‐RADS v2	
Negative	3 (2%)
3	16 (12%)
4	58 (45%)
5	53 (41%)
Diameter (mm), median (IQR)	14 (11, 17)
Location	
Anterior	94 (66%)
Posterior	49 (34%)
Laterality	
Left	82 (57%)
Right	60 (42%)
Unknown	1 (1%)
Grade group	
1 (MCCL ≥ 6 mm)	5 (4%)
2	92 (64%)
3	36 (25%)
4	10 (7%)
MCCL^b^ (mm), median (IQR)	7 (5, 9)
% Pattern 4, median (IQR)^c^	20 (10, 30)

^a^ Other race includes Latin American and other.

^b^ MCCL = maximum cancer core length.

^c^ Among the 92 patients with GG2.

### 2.3 Outcomes

The primary outcome measure was the result of MRGB six months after treatment. Absence of csPCa was considered a near‐term success, after which an 18‐month MRGB was scheduled to follow. Absence of csPCa on 18‐month MRGB was considered an intermediate‐term success, after which patients were included in an ongoing active surveillance program described previously. Small amounts of GG1 (<6 mm) were not considered csPCa.^22^, ^23^

Patients found to have csPCa on either follow‐up biopsy were considered to have reached a study endpoint at which time the men underwent surgery, radiation, repeat PGA,^24^ or expectant management (Figure 1). Serum PSA levels were obtained at baseline and throughout the follow‐up period. Other important outcomes were changes in urinary and sexual function assessed by EPIC‐Clinical Practice Questionnaire (EPIC‐CP) before and 6 months after treatment.^25^

### 2.4 Magnetic resonance imaging (MRI) and MRI‐guided biopsy (MRGB)

MRI and MRGB were performed at baseline (*n* = 143), 6 months (*n* = 136), and 18 months (*n* = 71). MRGB followed the imaging within one month at each time point. The MRI protocol and biopsy procedure have been detailed previously.^20^, ^26^ In brief, 3T multiparametric MRI was performed with an abdominal coil; interpretation was under supervision of a genitourinary radiologist with experience reading more than 5000 mpMRI studies (E.F.). Lesions seen on MRI were contoured, and the images were then transmitted electronically into an image‐fusion device (Artemis, Eigen Corp.). MRI‐ultrasound fusion for targeted and systematic sampling was performed by the device operator (L.M.); spatial location of all biopsy sites within the prostate was recorded automatically for tracked sampling. Method of biopsy sampling—targeted and systematic at baseline; targeted, tracked and systematic on treated‐side at 6 months; and targeted, tracked, and bilaterally systematic at 18 months—is shown in eFigure 1.

### 2.5 Treatment

Participants underwent partial gland cryoablation under general anesthesia via trans‐perineal 14‐gauge Argon cryoprobes (Galil Medical, Inc.) as previously described.^18^ Repeat treatments are not included and have been reported separately.^24^ Images from the biopsy screen‐capture were used in the operating room “cognitively” to guide cryoprobe placement (eFigure 2). Two or three probes were inserted unilaterally, so as to allow the iceball to incorporate the MRI lesion and positive biopsy locations. Two cycles of freezing were employed in all patients; safety monitoring was via real‐time imaging with ultrasound and thermal monitoring probes. All treatments were performed in the UCLA outpatient surgi‐center. A few hours after cryotherapy, patients were discharged with a Foley catheter in place and scheduled for a voiding trial at 48 h. A single dose of gentamycin was administered pretreatment, and one week of a quinolone antibiotic was used after treatment. The alpha‐blocker alfuzosin was used for one month postoperatively.

### 2.6 Follow‐up

Follow‐up visits were at 3, 6, 12, and 18 months post‐treatment. Serum PSA (Total and %Free) was obtained at each visit. MRGB was performed at baseline and at 6 and 18 months post‐treatment, as described above. Quality‐of‐life (QOL) outcomes were assessed with EPIC‐CP questionnaires, which were administered before treatment and 6 months afterward.^27^ Six patients, who underwent radical prostatectomy when follow‐up biopsy revealed csPCa, are described separately below.

### 2.7 Analysis

Clinical characteristics were summarized with frequencies for categorical data and means (SD) or medians (IQR) for continuous data. Clinical outcomes were analyzed as changes from baseline to 6 months, baseline to 18 months, and 6 to 18 months as paired data using Kappa test of agreement for categorical data; Wilcoxon signed rank sum test was used for continuous variables. A multivariate logistic regression was performed, looking for possible predictors of failure (converse of success, i.e., ≥GG2 or ≥6 mm GG1) at 6 and 18 months. A priori, we chose covariates to include in the model: age, prostate volume (≤40 cc vs 41–55 vs >55), PSA density (≤0.15 ng/mL/cc vs >0.15), lesion location (posterior vs anterior), lesion laterality (left vs left), maximum cancer core length (MCCL), pretreatment Grade Group, and year of procedure (2017–2019 vs 2020–2021).

## 3 RESULTS

Baseline clinical characteristics of the 143 men enrolled are shown in Table 1. The large majority of enrollees had Grade Group (GG) 2,3, or 4 pathology (*n* = 138, 96%); five had GG1 pathology with max cancer core length (MCCL) > 6 mm.^22^ All men were treatment‐naive, and stage was T1c (nonpalpable). Of the 143 patients, 35 (24%) had participated in active surveillance prior to enrollment; average duration in surveillance was 26 months, range 2 months to 9 years. The men all exhibited csPCa with the majority having GG2 cancers located in or near a lesion visible on MRI.

The cryotherapy procedure was completed successfully in all patients and required approximately 1 h of operating time. No serious intra‐operative complications occurred. All men were discharged as planned a few hours postoperatively. No bleeding or septic episodes were encountered.

### 3.1 Patient flow through trial

Of the 143 men enrolled in the trial, 136/143 (95%) completed a 6‐month biopsy and QOL questionnaires, as shown in the flow chart (Figure 1). At the 6‐month biopsy, 103/136 (76%) were found to have no csPCa on MRGB, and were termed initial or technical “successes.” Subsequently, 71 of the 103 underwent 18‐month MRGB; 46/71 (65%) were found to have no csPCa at that point, and were deemed intermediate‐term successes.

**FIGURE 2:**
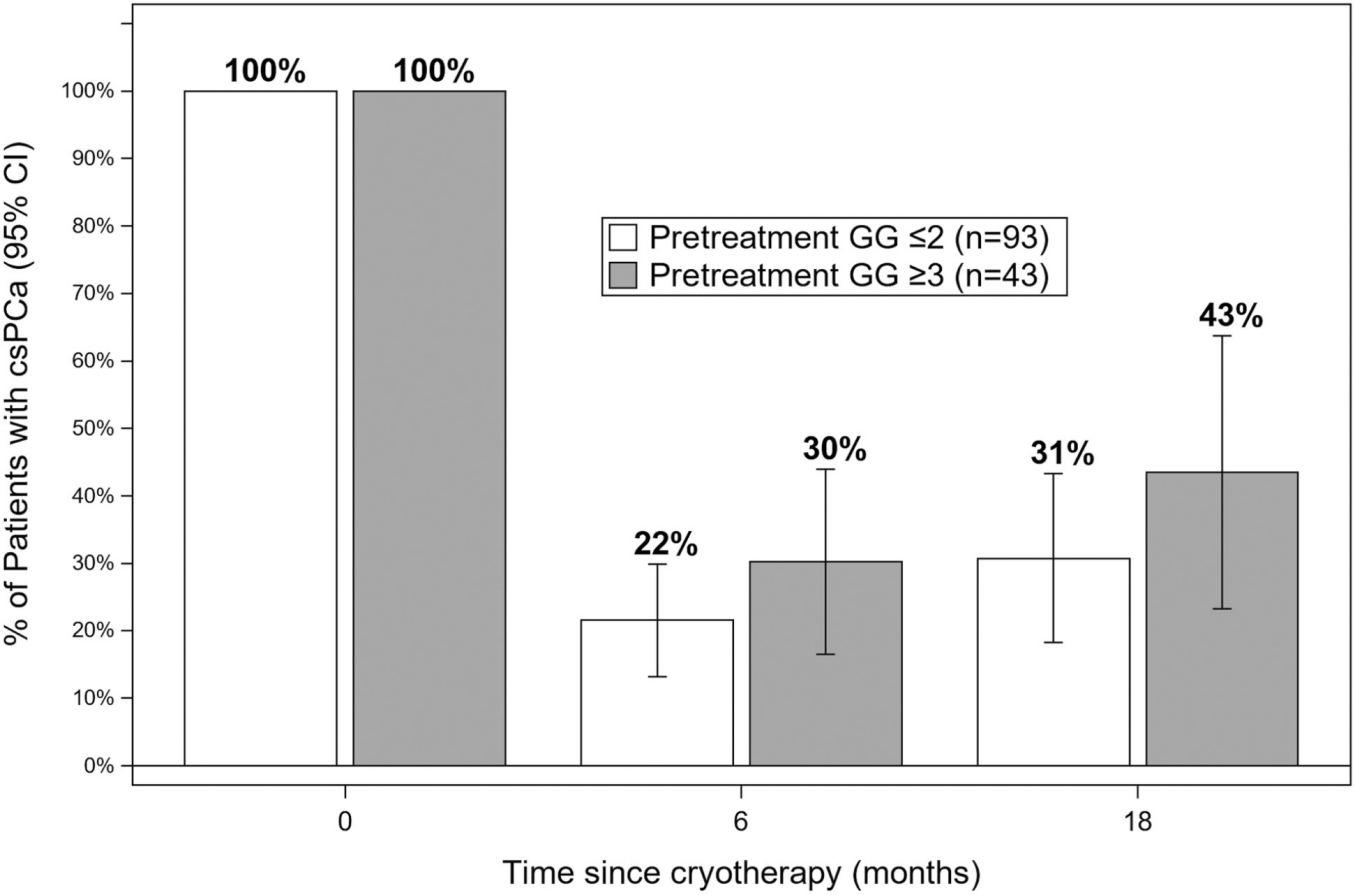
Probability of csPCa before and after partial gland ablation (PGA). Chart showing chances of csPCa on MRI‐guided biopsy (MRGB) before and after partial gland ablation (PGA) with cryotherapy. At baseline (time zero), all 136 patients had csPCa, either GG2 (open boxes, *N* = 93, including 5 with high‐volume GG1) or GG>3 (dark boxes, *N* = 43). Biopsy evidence of csPCa was found at 6 months in 22% of men whose pretreatment cancer was GG2 and in 30% of men whose cancer was >GG3. Among men with pretreatment cancer of GG3, 30% were found to have csPCa at 6 months and 43% at 18 months. Chances of a successful ablation, defined as absence of csPCa on MRGB, decreased somewhat over time, but were not significantly different between GG2 and GG3 groups. Overlap of the 95% C.I. is appreciable at both follow‐up intervals.

In Figure 2, histologic results of ablation are shown, comparing baseline biopsy, 6‐month biopsy, and among the 71 men with no csPCa at 6 months, 18‐month biopsy. In the figure, results for men with GG2 at baseline are shown in comparison with men having ≥GG3 at baseline. A successful ablation, that is, absence of csPCa, was found more often at both 6 and 18 months in men with GG2 than GG3 lesions, but the confidence intervals overlap considerably (*p* = 0.27 and 0.29, respectively). In each group, a successful ablation at 6 months was followed by a maintained success at 18 months in the majority of men.

### 3.2 Clinical and biopsy outcomes

In Table 2, details of clinical outcomes are shown for all men completing MRGB at 6 and 18 months after cryotherapy. At 6 months, success rate (no csPCa) was 76% (103/136). Among the failures (33/136, 24%), most were GG2 (*n* = 23) and nine were GG3/4. At 18 months, success rate was 65% (no csPCa in 46/71 men). Among the failures at 18 months (25/71, 35%), most cancers were GG2 (18 men) and some were GG3/4 (7 men). Ten men had GG4 lesions at baseline, and six of them were successes at 18 months. No metastatic PCa was diagnosed at either time point.

**TABLE 2:** Clinical outcomes (*n* = 136).

	Baseline (*n* = 136)	6 months (*n* = 136)	18 months (*n* = 71)	*p* ^a^
Grade group				
No Cancer	0 (0%)	89 (66%)	33 (46%)	NA
GG1	5 (4%)	14 (10%)	13 (18%)	
GG2	88 (65%)	24 (18%)	17 (24%)	
GG3	35 (26%)	7 (5%)	5 (7%)	
GG4	8 (6%)	2 (1%)	3 (4%)	
GG5	0 (0%)	0 (0%)	0 (0%)	
MCCL, mm, median (IQR)	7 (5, 9)	3 (2, 5)	3 (2, 5)	All <0.01
PSA, ng/mL				
Median (IQR)	6.9 (4.9, 9.9)	2.5 (1.3, 4.3)	2.5 (1.4, 4.1)	All <0.01
<4	13 (10%)	95 (70%)	53 (75%)	All <0.01
4–10	89 (65%)	39 (29%)	16 (23%)	
>10	34 (25%)	2 (1%)	2 (3%)	
Prostate volume, cc, median (IQR)	42.5 (33.4, 54.8)	34.9 (26.0, 45.0)	33 (25, 46)	<0.01, <0.01, 0.44
% Decrease	—	16.5 (5.6, 26.7)	‐3.2 (‐16.2, 8.0)	—
PSA Density, ng/mL/cc				
Median (IQR)	0.15 (0.12, 0.25)	0.06 (0.04, 0.11)	0.06 (0.04, 0.11)	<0.01, <0.01, 0.08
≤0.15	66 (49%)	113 (83%)	61 (87%)	<0.01, <0.01, 0.76
>0.15	69 (51%)	23 (17%)	9 (13%)	
% PSA decrease from baseline, median (IQR)	—	65% (40, 80)	57% (37, 75)	—
% Free PSA, mg/mL, median (IQR)	13 (10, 17)	16 (12, 22)	18 (12, 25)	<0.01, <0.01, 0.93

^a^ Kappa test of agreement or Wilcoxon signed rank sum test for ssbaseline vs 6 months, baseline vs 18 months, and 6 months vs 18 months.

Median PSA decreased significantly from 6.9 ng/mL at baseline to 2.5 ng/mL at the 6‐month interval (*p* < 0.01); further PSA decrease at 18 months was minimal (*p* = NS). A similar decrease from baseline to 6 months was also seen with prostate volume (approximately 20% volume reduction) and PSA density (*p* < 0.01), with further decreases at 18 months not significant (*p* = NS). Percent free PSA increased at 6 months (*p* < 0.01), then stabilized.

In Table 3 is shown the association of baseline characteristics with biopsy outcome at six and 18 months. At 6 months, where data were 95% complete, failure rate was significantly associated with baseline PIRADS score (*p* = 0.03), but not at 18 months (*p* = 0.27), where data were less complete. Men with posterior tumors failed more often at 18 months than men with anterior tumors (*p* = 0.03), and men treated in the 2nd half of the study were more likely to succeed at 18 months than men treated in the 1st half (86% vs 56%, *p* = 0.02) (data not shown). Age, prostate volume, and PSA levels were not associated with biopsy outcomes.

**TABLE 3:** Effects of baseline characteristics on treatment result (success vs failure; *n* = 136).

	6‐month		*p*	18‐month		*p*
	Success (*n* = 103)	Failure (*n* = 33)		Success (*n* = 46)	Failure (*n* = 25)	
Age						
50–65	44 (43%)	10 (30%)	0.41	21 (46%)	12 (48%)	0.96
66–75	41 (40%)	17 (52%)		20 (43%)	10 (40%)	
>75	18 (17%)	6 (18%)		54 (11%)	3 (12%)	
MCCL, mm, median (IQR)	7 (5, 9)	8 (5, 10)	0.07	7 (5,10)	7 (4,8)	0.36
PSA, ng/mL						
Median (IQR)	6.8 (4.9, 9.2)	7.2 (4.9, 13.0)	0.40	6.8 (4.1, 9.6)	6.6 (5.2, 8.9)	0.61
<4	11 (11%)	2 (6%)	0.08	10 (22%)	1 (4%)	0.07
4–10	71 (69%)	18 (55%)		25 (54%)	20 (80%)	
>10	21(20%)	13 (39%)		11 (24$)	4 (16%)	
Prostate volume, cc,						
Median (IQR)	35.8 (27.7, 47.9)	33 (24, 43)	0.11	49.5 (34, 58.8)	36 (31.8, 48)	**0.03**
≤40	44 (43%)	21 (64%)	0.11	18 (39%)	15 (60%)	**0.04**
41–55	32 (31%)	7 (21%)		12 (26%)	8 (32%)	
≤40	27 (26%)	5 (15%)		16 (35%)	2 (8%)	
PSA density, ng/mL/cc						
Median (IQR)	0.15 (0.12, 0.22)	0.16 (0.11, 0.39)	0.29	0.15 (0.08, 0.20)	0.19 (0.14, 0.27)	**0.03**
≤0.15	51 (50%)	15 (45%)	0.65	23 (50%)	10 (40%)	0.42
>0.15	51 (50%)	18 (55%)		23 (50%)	15 (60%)	
% Free PSA, median (IQR)^a^	17 (13, 23)	13 (10, 17)	**0.001**	13.5 (10, 18)	14 (11, 17)	0.86
PIRADS grade						
1–3	21 (21%)	2 (6%)	**0.03** ^b^	14 (31%)	5 (21%)	0.27^b^
4	48 (48%)	13 (39%)		19 (42%)	13 (54%)	
5	32 (32%)	18 (55%)		12 (27%)	6 (25%)	
ROI location						
Anterior	66 (64%)	22 (67%)	0.79	19 (41%)	4 (16%)	**0.03**
Posterior	37 (36%)	11 (33%)		27 (59%)	21 (84%)	
Laterality						
Left	61 (59%)	19 (58%)	0.87	27 (59%)	14 (56%)	0.83
Right	42 (41%)	14 (42%)		19 (41%)	11 (44%)	

*Note*: R0I diameter, which was the only significant baseline predictor of biopsy result in multivariate analysis, is treated in eTable 1.

^a^ 6‐month Success *n* = 93 vs Failure *n* = 31, 18‐month Success *n* = 38 vs Failure *n* = 22.

^b^ Wilcoxon rank sum test.

**FIGURE 3:**
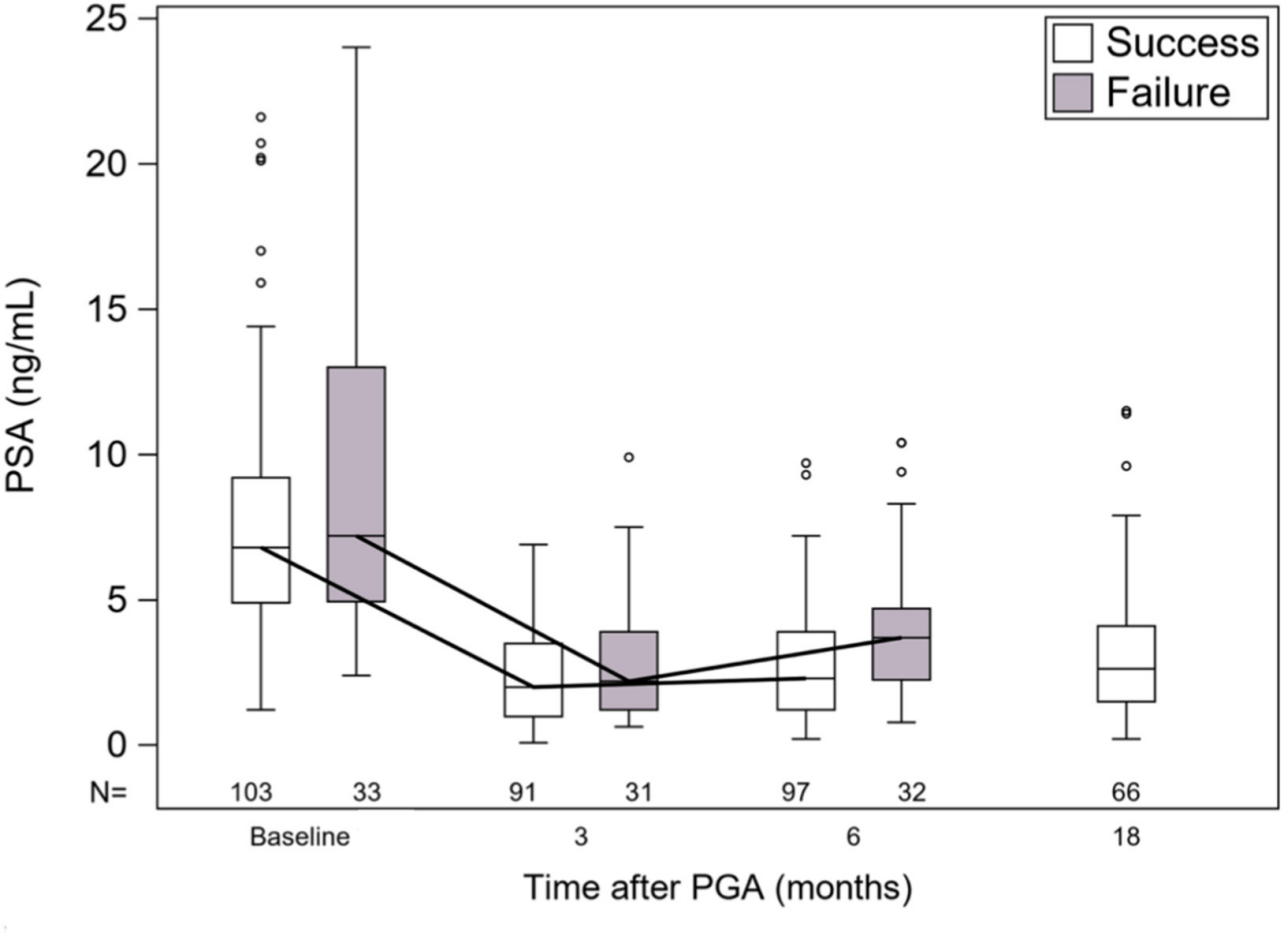
Effects of cryotherapy on serum PSA Levels. Box and Whisker plot showing serum PSA levels at baseline and subsequent time points following PGA with cryotherapy. At all time points, PSA levels in men who had a successful ablation (6‐mo biopsy no csPCa) were not substantially different from PSA levels in men failing cryoablation (6‐month biopsy csPCca). Patient data were censored at the 6‐month failure timepoint, and PSA data available for 66 men who succeeded at 6 months are displayed.

The relationship between serum PSA levels and biopsy outcomes is shown in Figure 3. PSA levels are comparable at baseline in men who later succeed or fail at 6 months. For failures and successes, PSA levels decline in parallel at 3 and 6 months, the two groups having comparable median values (*p* = NS) at the 6‐month biopsy. Among the 71 men, who succeeded at 6 months and continued in Active Surveillance, PSA levels at 18 months remained as at the 6‐month point. Men who were considered successes vs failures at 18 months (65% vs 35%) had comparable PSA levels at that time (data not shown). In eFigure 3 are shown chances of success vs percent decline of PSA levels following treatment.

### 3.3 MRI findings

MRI lesions, which were present at baseline in 133/136 patients (98%), were no longer apparent in 96/130 patients (74%) at the 6‐month point. Among the 96 with no lesions, 22 (23%) had csPCa; among the 34 with MRI lesions, 11 (32%) had csPCa (*p* = NS). Contralateral MRI lesions, appearing de novo at the 6‐month point, were seen in 10/130 (8%) patients at 6 months. Upon targeted biopsy of the new contralateral lesions, none contained csPCa.

Among the 71 men undergoing 18‐month biopsy, MRI showed a lesion in 25 (15 had csPCa) and no lesion in 46 (10 had csPCa) (*p* < 0.01). In total, csPCa was found in 25/71 (35%), of which 8 were ipsilateral, 12 were contralateral, and 5 were bilateral. In the 10 men with de novo MRI lesions at 18 months, two were ipsilateral (1 had csPCa), and 8 contralateral (4 had csPCa). In only 3/71 men was a high‐grade cancer found at 18 months (>GG4) (Table 2).

### 3.4 Multivariate analysis

Using the 6‐month data, a multivariate analysis was conducted to determine predictors of operative result (i.e., success vs failure upon follow‐up biopsy) (eTable 1). Of the variables analyzed, three emerged as statistically significant: age, prostate volume, and diameter of ROI, each of which was directly related to chance of failure. However, the width of the confidence intervals was large for age and volume. Thus, only ROI diameter was significantly predictive of treatment outcome, the chances of finding csPCa increasing 17% with each cm of ROI diameter (*p* < 0.02).

### 3.5 Urinary and sexual function

**FIGURE 4:**
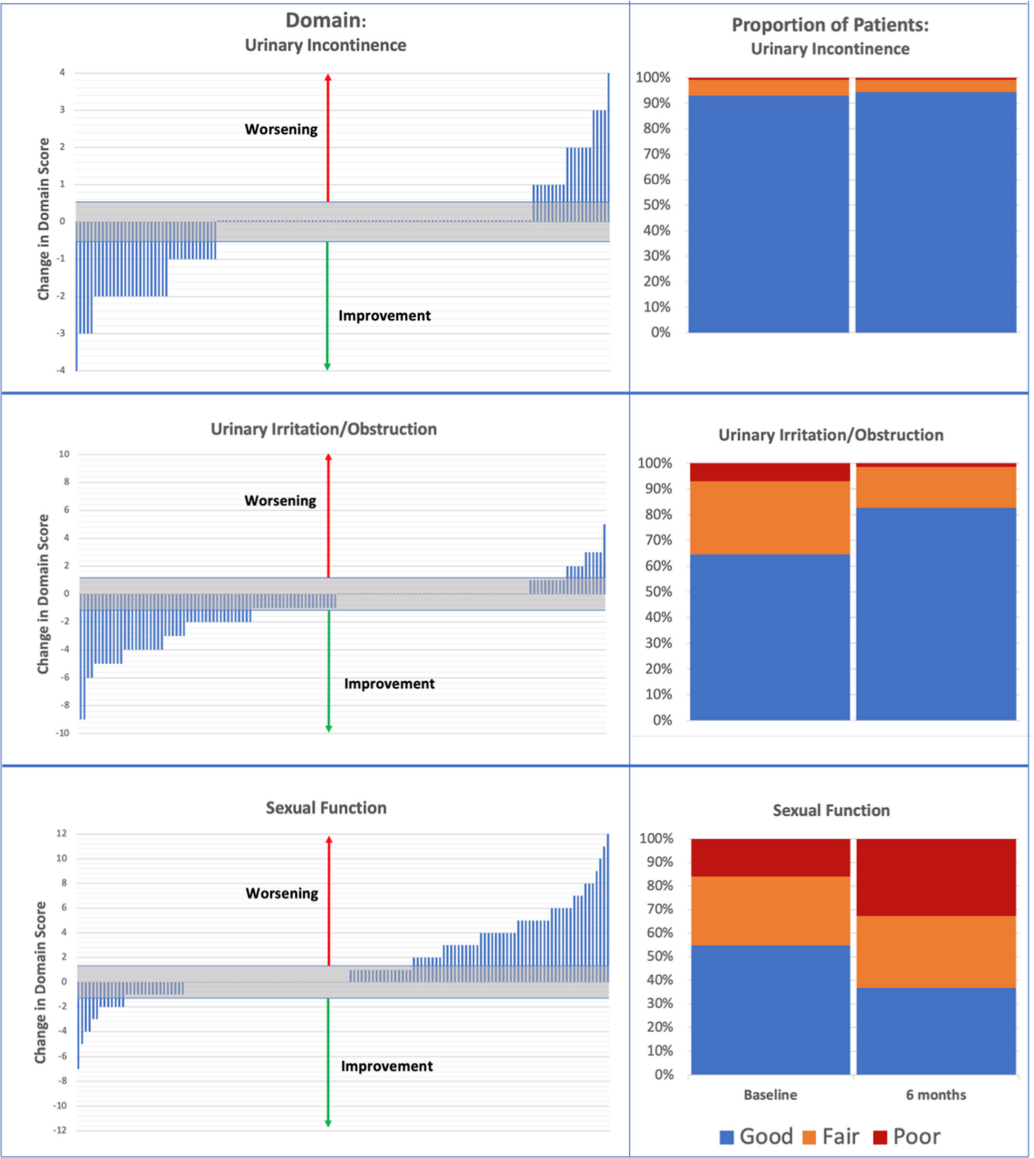
Effects of cryotherapy on urinary and sexual function. Waterfall plots (left) and group proportions (right) for all 143 men completing the EPIC‐CP questionnaires at baseline and 6 months. Three domains are analyzed: urinary incontinence (upper panels), urinary irritation/obstruction (middle panels), and sexual function (lower panels). In the waterfall plots, the band of clinical insignificance (change <0.5 SD) is shown in gray (shaded); upward bars indicate worsening, downward bars indicate improvement; middle area indicates no change. In stacked bars on right, blue = good (score 0–3), orange = fair (score 4–6), and red = poor (score 7+).^27^ Less than 10% of the group reported issues with continence before treatment, and this proportion declined slightly after treatment (upper panel). Approximately one‐third of the men reported obstructive/irritative symptoms before treatment (middle panel); this proportion decreased to approximately 20% after treatment. Conversely, regarding sexual function (lower panel), 83% of men reported good or fair sexual function before treatment; after treatment that proportion decreased to 70%.

In Figure 4, the effects of PGA on urinary and sexual function at the 6‐month interval, as indicated from responses on the EPIC‐CP questionnaire, are shown for all 143 men. In the waterfall plot, the incontinence domain worsened in 21 patients (15%); the irritative/obstructive domain worsened in 11 patients (8%); and the sexual function domain worsened in 53 patients (39%). Of the 53 reporting decreased overall sexual function, only 10 reported a severe decrement (>6 points increase in the domain score).^27^ Incidence and severity of urinary and sexual after‐effects were not related to pathologic outcome of cryotherapy (data not shown).

In general, the group data indicated that urinary symptoms were somewhat diminished and sexual symptoms moderately increased after treatment. Erection, orgasm, and overall sexual function were each affected similarly, according to the individual questionnaires (*p* = NS). Details are shown in Figure 4 and accompanying legend.

### 3.6 Radical prostatectomy following cryoablation

Six patients from the original group of 136 underwent radical prostatectomy, all following demonstration of residual or progressive PCa (Figure 1). Five of the six had GG3 lesions during the 18 months; upon final pathology, four were found to have locally extensive lesions (pT3). None of the 6 were found to have lymph node spread or metastases. Postoperative PSA levels were available in 5 of the men (8–30 months post‐op): 4 men have PSA <0.1 ng/mL and one is undergoing evaluation for a level of 0.5 ng/mL.

## 4 DISCUSSION

In the present trial, PGA by cryotherapy is evaluated using follow‐up MRGB as the determinant of success. Of men enrolling, 136/143 (95%) completed a follow‐up biopsy at six months and 71, who had no csPCa, then completed a second follow‐up biopsy at 18 months. The success rates, defined as absence of csPCa on MRGB, were 76% at six months and 65% at 18 months. No serious complications of the outpatient treatment were encountered; sexual and urinary after‐effects compared favorably with those reported after surgery or radiation.^28^ Thus, these data provide comprehensive biopsy evidence supporting the use of PGA‐cryotherapy as a relatively safe, moderately effective focal treatment of prostate cancer in the intermediate term.

Strengths of the study include the prospective design, the inclusion of only men with csPCa, and a strict protocol mandating MRGB before and after treatment with a 95% compliance. Biopsy was used to determine success or failure. Other studies have suggested PSA‐based “metrics of success” after various forms of PGA: PSA decrease from baseline (>80%),^29^ PSA nadir (<2.5 ng/mL),^10^ PSA increase from nadir (<1 or 2 ng/mL),^7^, ^8^, ^11^, ^30^ and PSA density (<0.15).^31^ Through 18 months of follow‐up after PGA‐cryotherapy, the present data failed to identify any PSA metric as a means to reliably identify individual success or failure when the determinant is biopsy outcome (Figure 3). Wysock and associates concluded similarly.^14^ In most men studied here, PSA levels declined, a low nadir was reached, and increases from nadir remained slight, regardless of biopsy outcome.

Dickinson suggested that disappearance of MRI lesions indicate a successful ablation,^31^ but in the present series, MRI lesions disappeared at 6 months in 74% of patients; biopsy outcome did not correlate with MRI findings. Conceivably, the treatment per se alters the internal architecture of the prostate, obscuring MRI detection of cancer. In follow‐up beyond the short‐term, MRI may regain a degree of sensitivity for cancer detection, as indicated by the 18‐month data reported here. However, in the near‐ and intermediate‐term, management decisions would seem best supported by biopsy findings. In a consensus statement, biopsy is recommended at follow‐up intervals, as provided in the present report.^32^

Potential sexual and urinary after‐effects are of major interest to men considering treatment for PCa. These after‐effects are reported for each of 143 men in a “waterfall” plot in the present report, using validated questionnaires (Figure 4).^33^ Urinary function generally remained unchanged, or it improved. Incontinence was very rare. In men reporting good or fair sexual function at baseline, treatment‐related decrements were severe in 10/143 men but for the great majority were generally mild or moderate. The present data confirm a substantially lesser impact on quality of life after PGA‐cryotherapy than that reported after surgery or radiation.^25^, ^28^

For men with intermediate‐risk PCa—as in 128/143 (90%) of the present patients—treatment options include surgery, radiation, PGA, or for men with GG2 lesions, active surveillance.^16^ Active surveillance is preferred for most men with low‐risk PCa (GG1 lesions), but for men with intermediate‐risk PCa (GG2), the risk of future upgrading during A.S. is appreciable.^34^ Results of the present study support the use of cryotherapy‐PGA for men with both GG2 and GG3 lesions, although the success rate in the GG3 group is somewhat lower than in the GG2 group.

PGA has comparatively few after‐effects, and at least in the intermediate term, it provides a reasonable chance of success, as shown here. At the 18‐month follow‐up, high‐grade disease was found in only 3/71 men, a rarity probably explained by the accuracy of the preceding MRI‐guided biopsies. Further, when PGA fails and prostatectomy is performed, results of delayed operation are found comparable to up‐front surgery.^35^, ^36^, ^37^ Operative results in our six men undergoing prostatectomy are supportive of that finding; a detailed evaluation of post‐treatment prostatectomy specimens will be the subject of a future report. Thus, PGA is increasingly chosen, even though long‐term benefit is not yet proven or quantified. The 35% incidence of csPCa at 18 months, as reported here, indicates need for continued surveillance. Relevant to the decision‐making process, Watson and colleagues showed that many men with intermediate‐risk prostate cancer are willing to trade a certain amount of length for preservation of quality years of life.^38^

Limitations of the present study include the relatively brief duration of follow‐up and the nonrandomized design. MRI interpretation in treatment‐naive patients is standardized, but here the same system was used after treatment, where consensus guidelines are not available.^39^ Further, while biopsy follow‐up was 95% complete at 6 months, the primary endpoint (showing a 76% success rate), a second follow‐up biopsy at 18 months was only obtained in 69% of men (showing a 65% success rate). The 6‐ and 18‐month biopsies were chosen to provide men, who failed PGA‐cryotherapy, early treatment options before disease progression could occur and subsequently, to determine whether, at an intermediate time point, the natural history of the disease was altered. In the present study, both goals were met (although only 71 men reached the secondary endpoint).

No comparison with high‐intensity focused ultrasound (HIFU), which has been practiced at our institution for more than a decade, is made here.^40^ However, when comprehensive MRGB is used to evaluate results of PGA with HIFU, outcomes appear similar to those reported here with cryotherapy.^41^ The two modalities are useful in different situations and are considered complementary methods of PGA.

A definitive statement regarding long‐term efficacy—and how “efficacy” should be defined for men with intermediate‐risk PCa—awaits lengthy follow‐up. Moreover, a recent study from Oxford University raises the possibility that cancer genomics (“unappreciated molecular relationships between histologically benign and cancerous regions”) may disrupt the current focal‐therapy paradigm.^42^ A randomized trial CHRONOS, now in early stages in England, is expected by 2027 to provide Level 1 evidence regarding PGA of prostate cancer.^43^

## AUTHOR CONTRIBUTIONS

**Mamdouh Aker:** Conceptualization (lead); data curation (lead); formal analysis (equal); investigation (equal); methodology (equal); project administration (equal); resources (equal); software (equal); supervision (equal); validation (equal); visualization (equal); writing – original draft (lead); writing – review and editing (lead). **Wayne Brisbane:** Conceptualization (lead); data curation (equal); formal analysis (equal); funding acquisition (equal); investigation (equal); methodology (equal); project administration (equal); resources (equal); software (equal); supervision (equal); validation (equal); visualization (equal); writing – original draft (equal); writing – review and editing (equal). **Lorna Kwan:** Conceptualization (equal); data curation (equal); investigation (equal); software (lead); writing – review and editing (equal). **Samantha Rose Gonzalez:** Formal analysis (equal); methodology (equal); project administration (equal); resources (equal); software (equal); writing – review and editing (equal). **Alan Priester:** Formal analysis (supporting); investigation (supporting); methodology (equal); validation (equal); visualization (equal). **Adam Kinnaird:** Investigation (equal); resources (equal); visualization (equal). **Merdie Delfin:** Project administration (equal); supervision (equal). **Ely R Felker:** Data curation (equal); formal analysis (equal); investigation (equal); validation (equal). **Anthony Sisk:** Data curation (equal); formal analysis (equal); investigation (equal); methodology (equal). **David Kuppermann:** Formal analysis (equal); investigation (equal). **Leonard S. Marks:** Conceptualization (equal); data curation (equal); formal analysis (equal); funding acquisition (lead); investigation (equal); methodology (equal); project administration (lead); resources (equal); supervision (lead); validation (equal); visualization (equal); writing – original draft (equal); writing – review and editing (equal).

## CONFLICT OF INTEREST STATEMENT

Dr. Marks is co‐founder, Avenda Health; Dr. Priester is employed as data scientist for Avenda Health.

## Supporting information


Figures S1.

Data S1.

## Data Availability

The data that support the findings of this study are available from the corresponding author upon reasonable request.
