# Supplementary material for: Cryotherapy for partial gland ablation of prostate cancer: Oncologic and safety outcomes
Source: Cancer Med. 2023 Feb 12;12(8):9351–62. doi: 10.1002/cam4.5692 (PMC10166973; doi:10.1002/cam4.5692)
Supplement: Supplementary file 1 — Figures S1. [file CAM4-12-9351-s001.docx]

eFigure 1. BIOPSY SCHEMA

**Baseline**

**6 Months**

**18 Months**

Biopsy schema employed during trial. At baseline (A), diagnosis was made by obtaining 3-5 targeted cores (green) from MRI lesions >PIRADS-3 (red inset) and systematic cores (blue) from a 12-point template. At 6-month follow-up after cryoablation (B), targeted cores (green) were obtained from the site of the original lesion (faded inset) and systematically from the ipsilateral side (blue). At 18-month follow-up (C), targeted cores (green) were obtained from the site of the original lesion (faded inset) and using template guidance, systematically from both sides (blue) (total 18-20 cores). Any new lesions appearing at 6- or 18- month biopsy were also sampled. Figure reproduced with permission from Chuang *et al*.^19^

Biopsy was performed transrectally in a urology clinic, using 1% xylocaine anesthesia, a spring-loaded gun, and an 18ga needle.^26^ Antibiotic prophylaxis consisted of one gram of ertapenem administered by intramuscular injection an hour before biopsy. All biopsies were performed by a single urologist (L.M.), and pathologic processing and interpretation was under supervision of a genitourinary pathologist (A.S.).


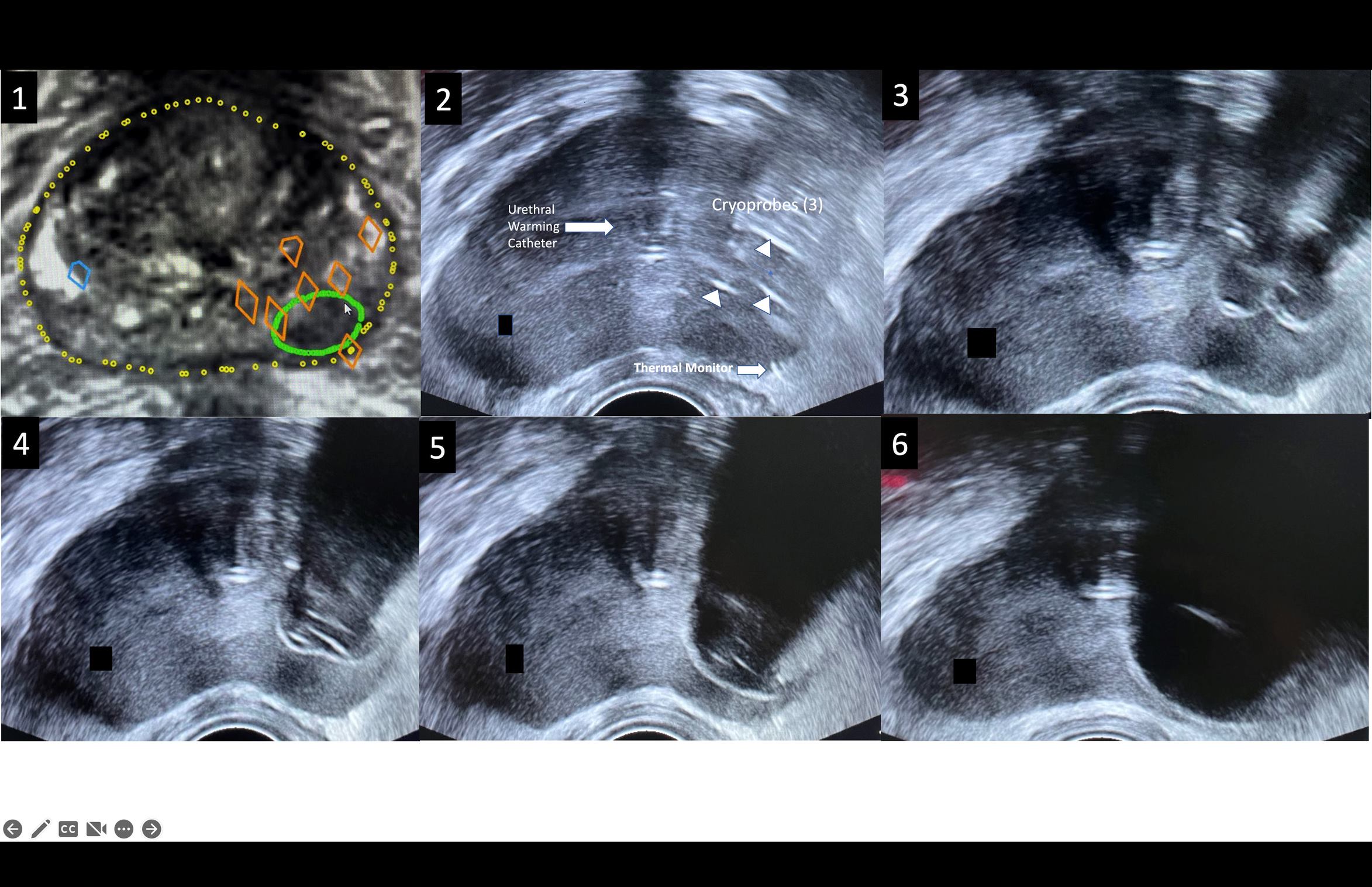
eFigure 2. CRYOTHERAPY PROCEDURE

Prostate images showing progress of a cryotherapy procedure. (1) From fusion biopsy, prostate outline is shown in yellow, MRI lesion in green, and cancer cores in orange. (2) Operation begins with placement of cryoprobes (arrow heads), thermal monitor, and urethral warming catheter. (3) Cryoprobes are activated, and (4-6) leading edge of ice ball appears. Ice ball extends posteriorly; treatment is stopped when tumor area is enveloped by ice ball and rectal wall is spared.

eFigure 3. PSA DECLINE vs HISTOPATHOLOGIC SUCCESS


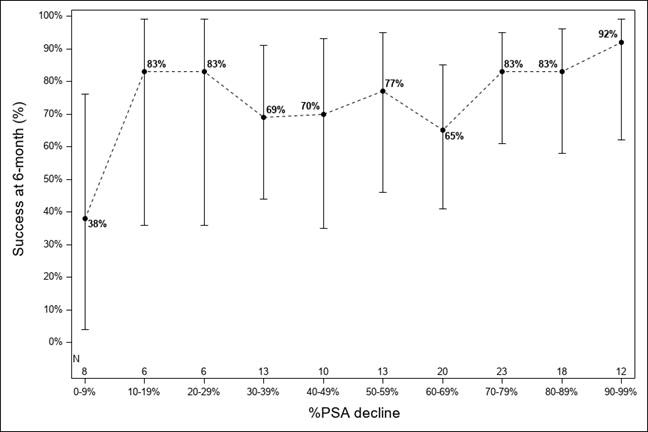


Frequency distribution showing percent chance of success on vertical axis vs percent decline of PSA from baseline on the horizontal axis. Data are shown as proportions and confidence intervals. Upon 6-month biopsy, participants whose PSA declined less than 10% had a greater chance of a positive biopsy than men whose PSA declined more than 10% (63% vs 22%; O.R. 5.8, p=.03) (left side of curve). Participants whose PSA declined more than 70% had a greater chance of a negative biopsy than those whose PSA declined less than 70% (32% vs 15%; O.R. 2.6, p=0.02) (right side of curve). Statistical significance was only present at the extremes of the curve.

eTable 1. Multivariate Analysis of Treatment Failure (n=136).

|  | OR (95% CI) | P |
| --- | --- | --- |
| Age at Cryotherapy (50-65 as referent) |  | 0.0664 |
| 66-75 | 3.92 (1.24, 12.41) |  |
| >75 | 1.93 (0.45, 8.21) |  |
| Prostate Volume, cc (>55 as referent) |  | 0.0155 |
| 41-55 | 1.24 (0.27, 5.69) |  |
| ≤40 | 6.13 (1.40, 26.91) |  |
| PSA density, ng/mL/cc (>0.15 vs ≤0.15) | 0.35 (0.11, 1.12) | 0.0760 |
| PIRADS Grade (0-3 as referent) |  | 0.6704 |
| 4 | 2.41 (0.35, 16.51) |  |
| 5 | 2.08 (0.27, 16.27) |  |
| ROI Location |  |  |
| Anterior (posterior vs anterior) | 1.80 (0.64, 5.08) | 0.2664 |
| Laterality (left vs right) | 1.24 (0.45, 3.42) | 0.6726 |
| ROI Diameter (cm) | 1.17 (1.03, 1.32) | 0.0159 |
| Baseline Grade Group (≤2 as referent) |  | 0.3754 |
| GG3 | 2.11 (0.70, 6.37) |  |
| GG4 | 2.01 (0.24, 17.09) |  |
| Year of Cryotherapy (2017-19 vs 2020-21) | 1.17 (0.42, 3.29) | 0.7677 |
|  |  |  |
