# Supplementary material for: Cryotherapy for partial gland ablation of prostate cancer: Oncologic and safety outcomes
Source: Cancer Med. 2023 Feb 12;12(8):9351–62. doi: 10.1002/cam4.5692 (PMC10166973; doi:10.1002/cam4.5692)
Supplement: Supplementary file 2 — Data S1. [file CAM4-12-9351-s002.pdf]

Help Live Chat Leonard Marks

Hemigland Cryoablation of Clinically Significant Prostate Cancer: Intermediate-Term Followup via Magnetic Resonance Imaging Guided Biopsy

Author: Ryan Chuang, Adam Kinnaird, Lorna Kwan, et al

Publication: Journal of Urology

Publisher: Wolters Kluwer Health, Inc.

Date: Sep 28, 2020

Copyright © 2020, Wolters Kluwer Health

Order Completed

Thank you for your order.

This Agreement between Leonard S Marks ("You") and Wolters Kluwer Health, Inc. ("Wolters Kluwer Health, Inc.") consists of your order details and the terms and conditions provided by Wolters Kluwer Health, Inc. and Copyright Clearance Center.

License number	Reference confirmation email for license number
License date	Nov, 10 2022

Licensed Content

Licensed Content Publisher	Wolters Kluwer Health, Inc.
Licensed Content Publication	Journal of Urology
Licensed Content Title	Hemigland Cryoablation of Clinically Significant Prostate Cancer: Intermediate-Term Followup via Magnetic Resonance Imaging Guided Biopsy
Licensed Content Author	Ryan Chuang, Adam Kinnaird, Lorna Kwan, et al
Licensed Content Date	Sep 28, 2020
Licensed Content Volume	204
Licensed Content Issue	5

Order Details

Type of Use	Journal/Magazine
Requestor type	STM Signatory Publisher
STM publisher name	Wiley
Format	Print and electronic
Portion	Figures/tables/illustrations
Number of figures/tables/illustrations	1
Author of this Wolters Kluwer article	Yes
Will you be translating?	No
Intend to modify/change the content	No
Publishing Open Access	Yes
Creative Commons License	CC-BY

About Your Work

**Title of new article**	Cryotherapy for Partial Gland Ablation of Prostate Cancer
**Lead author**	M. Aker
**Title of targeted journal**	Cancer
**Publisher**	Wiley
**Expected publication date**	Aug 2023

Additional Data

**Order reference number**	501770748
**Portions**	Figure 1, page 942, Journal of Urology Vol 204 (5), 2020

Requestor Location

**Requestor Location**	Leonard S Marks<br>924 Westwood Blvd<br>Suite 520<br><br>Los Angeles, CA 90095<br>United States<br>Attn: Leonard S Marks

Tax Details

**Publisher Tax ID**	13-2932696

Billing Information

**Billing Type**	Invoice
**Billing address**	Leonard S Marks<br>924 Westwood Blvd<br>Suite 520<br><br>Los Angeles, CA 90095<br>United States<br>Attn: Leonard S Marks

Price

**Total**	0.00 USD

Total: 0.00 USD

CLOSE WINDOW

© 2022 Copyright - All Rights Reserved | Copyright Clearance Center, Inc. | Privacy statement | Data Security and Privacy | For California Residents | Terms and ConditionsComments? We would like to hear from you. E-mail us at customercare@copyright.com
